# Supplementary material for: Dynamically cultured, differentiated bovine adipose-derived stem cell spheroids as building blocks for biofabricating cultured fat
Source: Nat Commun. 2024 Oct 22;15:9107. doi: 10.1038/s41467-024-53486-w (PMC11496621; doi:10.1038/s41467-024-53486-w)
Supplement: Supplementary file 1 — Supplementary Information file [file 41467_2024_53486_MOESM1_ESM.pdf]

Supplementary Information

Supplementary Table 1: Cell yield per body region. Cell yield when cells are extracted from fat from the chest, thighs, abdomen and back of 2-year-old Limousin cattle.

Isolated cells per gram fat			
Chest	Thigh	Abdomen	Back
122914.457	282131.661	161312.849	364552.656
468579.731	452378.742	277560.157	132859.829
277777.778	366550.377	52086.4878	166020.672
51785.5942	122699.313		387595.881
709731.078	186477.224		31090.1394
262579.653	314354.747		306231.779
284251.685	134121.871		
599552.334	182861.241		
734146.829	221801.138		
mean	mean	mean	mean
390146.571	251486.257	163653.165	231391.826
SD	SD	SD	SD
249120.431	111055.38	112755.052	142713.167

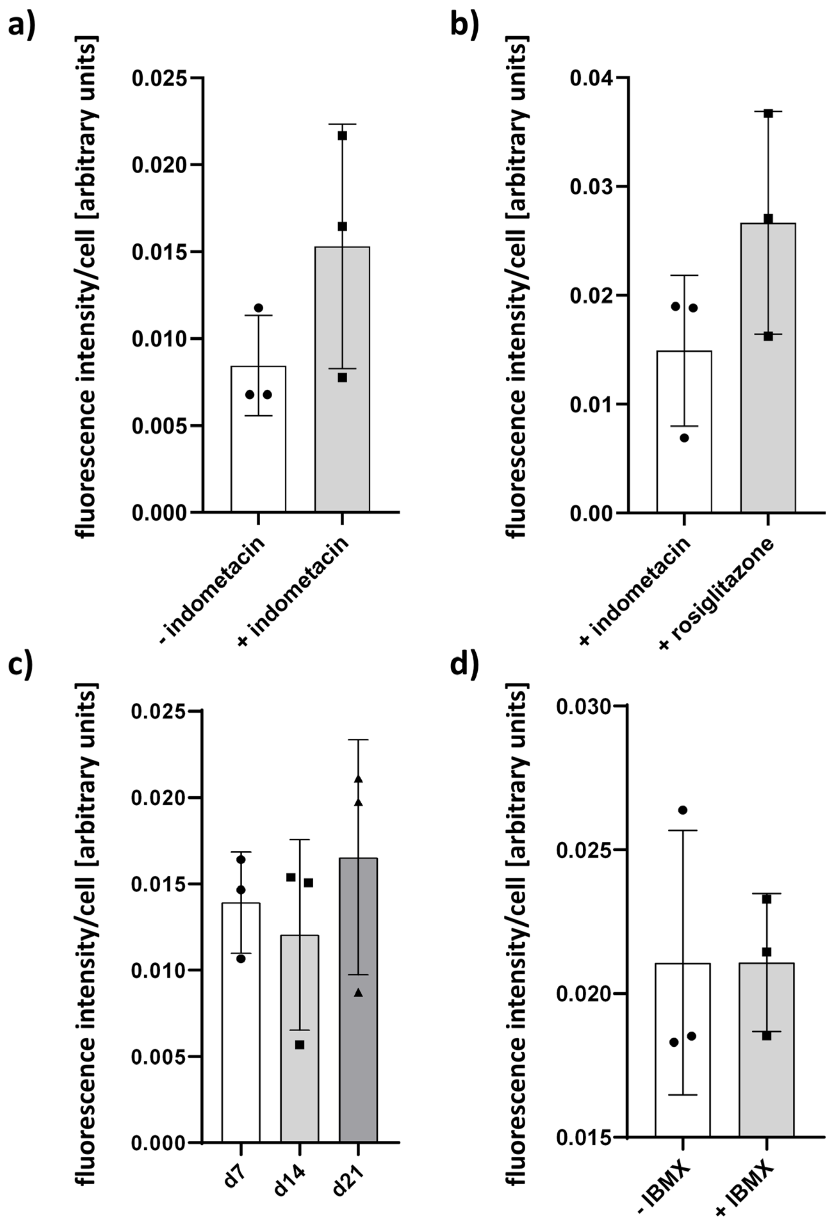

Supplementary Figure 1: Quantification of lipid accumulation in differentiated bASCs. a: Day 14 of 2D bASC differentiation with and without indomethacin and quantification of fluorescence signal/cell after BODIPY staining. DMEM-Diff - rosiglitazone + 3-Isobutyl-1-methylxanthin (IBMX); **b:** Day 14 of 2D bASC differentiation indomethacin vs rosiglitazone and quantification of fluorescence signal/cell after BODIPY staining. DMEM-Diff + IBMX; **c:** 2D bASC differentiation over time until day 21 and quantification of fluorescence signal/cell after BODIPY staining. DMEM-Diff + IBMX; **d:** Day 14 of 2D bASC differentiation with and without IBMX present in DMEM-Diff and quantification of fluorescence signal/cell after BODIPY staining; dots represent single data points; n=3 biological replicates. Data are plotted as mean and ± SD. Source data are provided as a Source Data file.

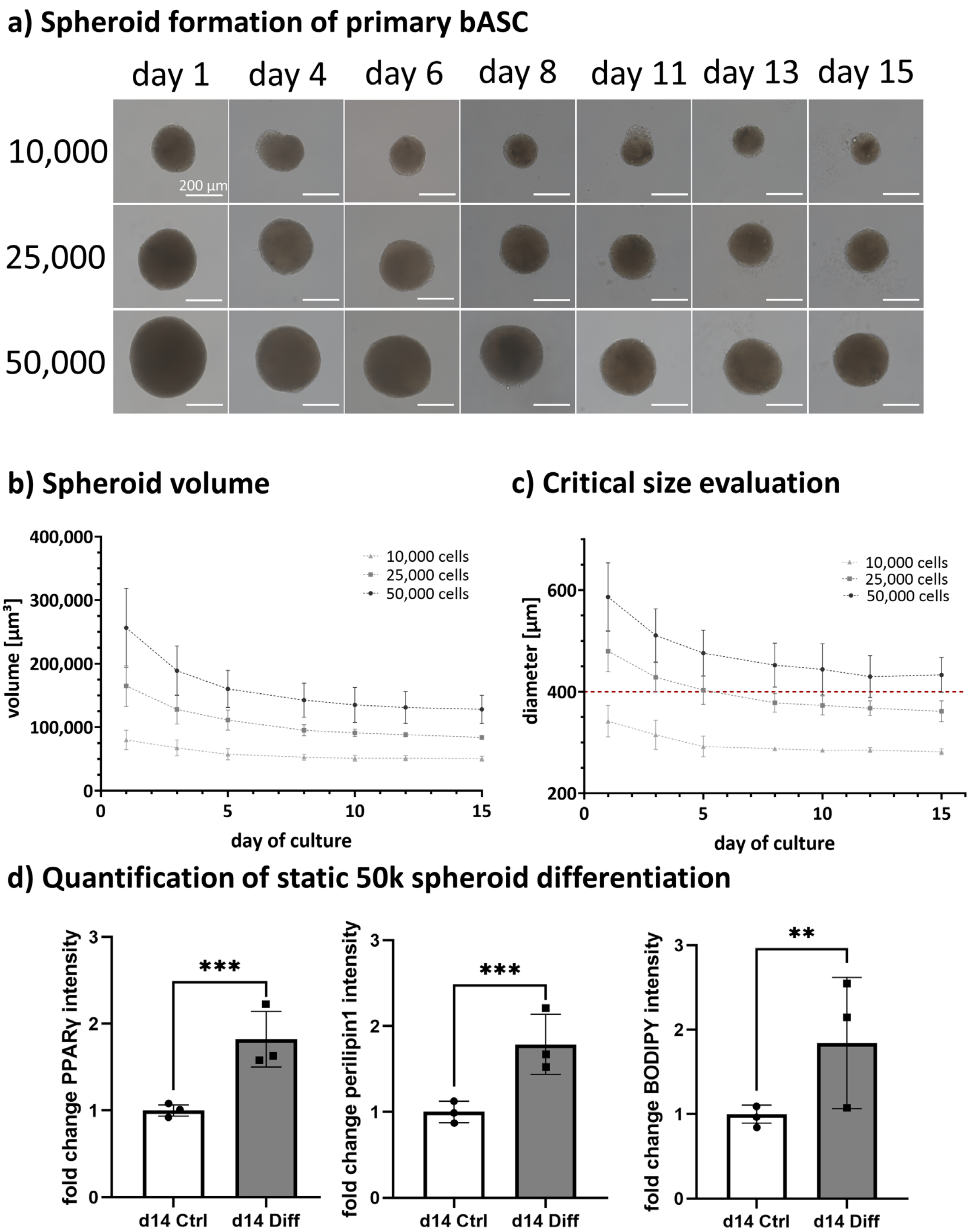

Supplementary Figure 2: Characteristics of bASC spheroids. **a:** Light microscopic images of 10,000, 25,000, and 50,000 cells per spheroid over 15 days. Scale bar: 200 µm; **b:** Determined spheroid volume over 15 days of maintenance.; **c:** Evaluation of the critical size limited by oxygen diffusion limit (marked red line); n=3; **d**: Quantified marker intensities of PPARγ ($p<0.001$), perilipin1 ($p<0.001$) and BODIPY ($p=0.005$) signals normed to day 14 control of 50k spheroids; Significances were calculated using Welchs t test; *: $p \leq 0.05$, **: $p \leq 0.01$, *** $p \leq 0.001$; n=3 biological replicates. Data are plotted as mean and ± SD. Source data are provided as a Source Data file.

a) Quantification of dynamic spheroid differentiation

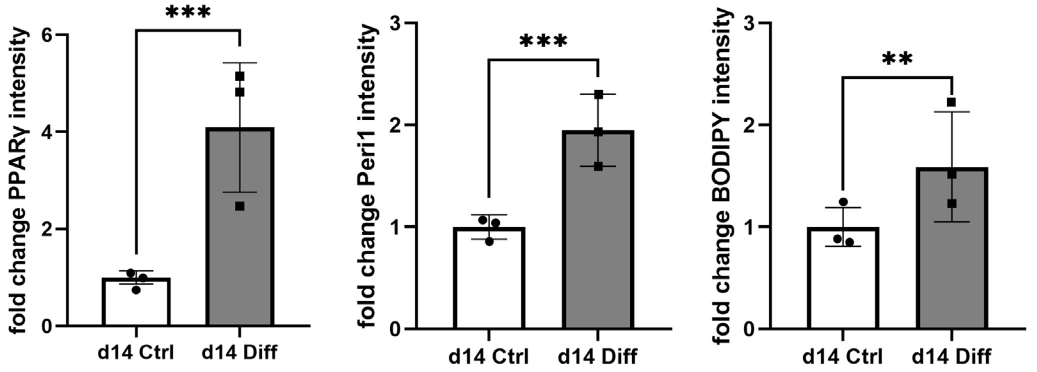

b) Zoom on printed grid

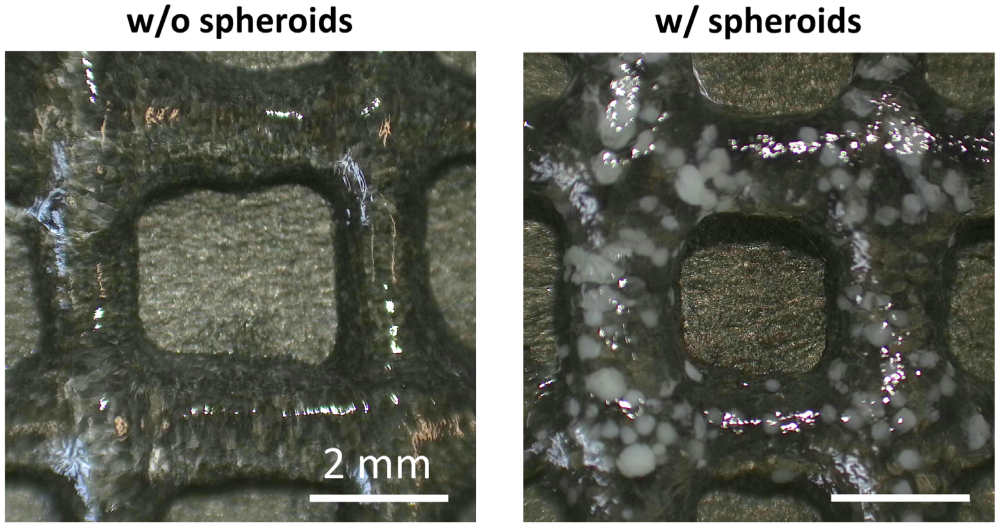

Supplementary Figure 3: Quantification and printing of dynamic spheroids. a: Quantification of marker intensity for dynamic spheroid staining. Marker intensities of PPARγ ($p<0.001$) perilipin1 ($p<0.001$) and BODIPY ($p=0.002$) signals normed to day 14 control; Significances were calculated using Welchs t test; *: $p \leq 0.05$, **: $p \leq 0.01$, *** $p \leq 0.001$; n=3 biological replicates. Data are plotted as mean and ± SD **b:** Zoom on printed grids of 1.5 % GG without (w/o) or with (w/) differentiated spheroids. Scale bar: 2 mm. Source data are provided as a Source Data file.

Supplementary Table 2: Composition of fatty acids in native bovine fat tissue, differentiated spheroids and control spheroids. Values for saturated (SFA), monounsaturated (MUFA) and polyunsaturated (PUFA) fatty acids in percent of all fatty acids. n = 3 biological replicates.

In % of total fat	Fat tissue			Differentiated spheroids			Control spheroids		
SFA	77	88.45	81	61.6	60.8	76.15	81.8	81.55	88.25
MUFA	21.55	10.1	17.75	33.85	34.15	13.8	6.15	5.9	5.4
PUFA	1.25	1.15	1.1	3.8	4.4	9.35	11.5	11.75	5.9
