# Supplementary material for: Dynamically cultured, differentiated bovine adipose-derived stem cell spheroids as building blocks for biofabricating cultured fat
Source: Nat Commun. 2024 Oct 22;15:9107. doi: 10.1038/s41467-024-53486-w (PMC11496621; doi:10.1038/s41467-024-53486-w)
Supplement: Supplementary file 2 — Reporting Summary [file 41467_2024_53486_MOESM2_ESM.pdf]

nature portfolio

Corresponding author(s): Petra Kluger

Last updated by author(s): Sep 16, 2024

Reporting Summary

Nature Portfolio wishes to improve the reproducibility of the work that we publish. This form provides structure for consistency and transparency in reporting. For further information on Nature Portfolio policies, see our Editorial Policies and the Editorial Policy Checklist.

Statistics

For all statistical analyses, confirm that the following items are present in the figure legend, table legend, main text, or Methods section.

n/a	Confirmed	
[ ]	[x]	The exact sample size (*n*) for each experimental group/condition, given as a discrete number and unit of measurement
[ ]	[x]	A statement on whether measurements were taken from distinct samples or whether the same sample was measured repeatedly
[ ]	[x]	The statistical test(s) used AND whether they are one- or two-sided<br>*Only common tests should be described solely by name; describe more complex techniques in the Methods section.*
[x]	[ ]	A description of all covariates tested
[ ]	[x]	A description of any assumptions or corrections, such as tests of normality and adjustment for multiple comparisons
[ ]	[x]	A full description of the statistical parameters including central tendency (e.g. means) or other basic estimates (e.g. regression coefficient) AND variation (e.g. standard deviation) or associated estimates of uncertainty (e.g. confidence intervals)
[ ]	[x]	For null hypothesis testing, the test statistic (e.g. *F*, *t*, *r*) with confidence intervals, effect sizes, degrees of freedom and *P* value noted<br>*Give P values as exact values whenever suitable.*
[x]	[ ]	For Bayesian analysis, information on the choice of priors and Markov chain Monte Carlo settings
[x]	[ ]	For hierarchical and complex designs, identification of the appropriate level for tests and full reporting of outcomes
[x]	[ ]	Estimates of effect sizes (e.g. Cohen's *d*, Pearson's *r*), indicating how they were calculated

Our web collection on statistics for biologists contains articles on many of the points above.

Software and code

Policy information about availability of computer code

Data collection	ZEN 2.3 blue (ZEISS, Carl Zeiss Microscopy, Germany)
Data analysis	ImageJ FIJI 1.53c Java 1.8.0_172 supported by Read_And_Write_Excel (GitHub, version 1.1.6)<br>GraphPad Prism 10.0.0<br>MATLAB (version R2022a, MathWorks, Natick, USA) with custom script deposited under: https://github.com/harkonsen/BODIPY

For manuscripts utilizing custom algorithms or software that are central to the research but not yet described in published literature, software must be made available to editors and reviewers. We strongly encourage code deposition in a community repository (e.g. GitHub). See the Nature Portfolio guidelines for submitting code & software for further information.

Data

Policy information about availability of data

All manuscripts must include a data availability statement. This statement should provide the following information, where applicable:

- Accession codes, unique identifiers, or web links for publicly available datasets
- A description of any restrictions on data availability
- For clinical datasets or third party data, please ensure that the statement adheres to our policy

All data generated or analyzed during this study are included in the published article and supplementary file. Source data are provided with this paper.

Research involving human participants, their data, or biological material

Policy information about studies with human participants or human data. See also policy information about sex, gender (identity/presentation), and sexual orientation and race, ethnicity and racism.

Reporting on sex and gender	N/A
Reporting on race, ethnicity, or other socially relevant groupings	N/A
Population characteristics	N/A
Recruitment	N/A
Ethics oversight	N/A

Note that full information on the approval of the study protocol must also be provided in the manuscript.

Field-specific reporting

Please select the one below that is the best fit for your research. If you are not sure, read the appropriate sections before making your selection.

[x] Life sciences   [ ] Behavioural & social sciences   [ ] Ecological, evolutionary & environmental sciences

For a reference copy of the document with all sections, see nature.com/documents/nr-reporting-summary-flat.pdf

Life sciences study design

All studies must disclose on these points even when the disclosure is negative.

Sample size	We did not use any statistical methods to predetermine sample size. Sample size was chosen based on similiar studies in this field. All experiments were performed at least 3 times with at least 3 technical replicates each time. The exact numbers are described in each figure legend.
Data exclusions	no exclusions
Replication	All findings can be reproduced but vary in quality between donor animals. All experiments were performed at least 3 times to ensure reproducibility.
Randomization	Cell culture experiments were performed with primary cells in passage 4. A fraction of the cell population was randomly allocated to differentiation treatments.
Blinding	Experimental data were collected and analyzed by multiple operators. Image acquisition of the samples was not performed with blinded samples. Other quantitative data were also obtained non-blinded, using automated or semi-automated methods independent of human bias.

Reporting for specific materials, systems and methods

We require information from authors about some types of materials, experimental systems and methods used in many studies. Here, indicate whether each material, system or method listed is relevant to your study. If you are not sure if a list item applies to your research, read the appropriate section before selecting a response.

Materials & experimental systems

n/a	Involved in the study	
[ ]	[x]	Antibodies
[x]	[ ]	Eukaryotic cell lines
[x]	[ ]	Palaeontology and archaeology
[x]	[ ]	Animals and other organisms
[x]	[ ]	Clinical data
[x]	[ ]	Dual use research of concern
[x]	[ ]	Plants

Methods

n/a	Involved in the study	
[x]	[ ]	ChIP-seq
[x]	[ ]	Flow cytometry
[x]	[ ]	MRI-based neuroimaging

Antibodies

Antibodies used	Primary antibodies: CD56 (1:50; 304602, BioLegend, San Diego, CA, USA), CD73 (1:50; 12231-1-AP, Proteintech Germany GmbH, Planegg-Martinsried, Germany), CD90 (1:50; 66766-1-Ig, Proteintech Germany GmbH), CD105 (1:50; 10862-1-AP, Proteintech

Germany GmbH), perilipin A (1:200; Ab 3526 Abcam, Cambridge, UK), PPAR Gamma (1:200; 16643-1-AP, Proteintech Germany GmbH), cCas3 (1:400, 9661 Cell Signaling), RIPK3 (1:200, LS-C354158, LifeSpan BioSciences Inc.), and Hif-1α (1:50; 14-9100-80, Invitrogen).

Secondary antibodies: anti-mouse (1:200; Ab150113) or anti-rabbit AlexaFluor 488(1:200, Ab150077, both Abcam, Cambridge, UK), anti-mouse Cy3TM3 IgG (1:200, 115165003, Dianova GmbH, Hamburg, Germany), and IgG control antibodies for monoclonal mouse IgG1 (Sc-3877) and IgG2a (Sc-3878, both Santa Cruz Biotechnology) and polyclonal rabbit IgG (NI01, Merck KGaA).

Validation

https://www.biolegend.com/en-gb/products/purified-anti-human-cd56-ncam-antibody-1607
https://www.ptglab.com/de/products/NT5E,CD73-Antibody-12231-1-AP.htm
https://www.ptglab.com/de/products/CD90-Antibody-66766-1-Ig.htm
https://www.ptglab.com/de/products/ENG-Antibody-10862-1-AP.htm
https://www.abcam.com/products/primary-antibodies/perilipin-1-antibody-ab3526.html
https://www.cellsignal.com/datasheet.jsp?productId=9661&images=1&_requestid=2072232
https://www.lsbio.com/antibodies/mprip-antibody-rip3-antibody-internal-ihc-wb-western-ls-c354158/365279
https://www.fishersci.de/shop/products/hif-1-mouse-clone-esee122-ebioscience/p-7069608

Plants

Seed stocks

N/A

Novel plant genotypes

N/A

Authentication

N/A
